# Ureteroscopic Lithotripsy

**DOI:** 10.1155/DTE.4.1

**Published:** 1997

**Authors:** Demetrius H. Bagley

**Affiliations:** Department of Urology Jefferson Medical College Thomas Jefferson University Philadelphia Pennsylvania USA

## Abstract

There is a wide array of endoscopic lithotriptors presently available. Each of these has its own advantages and disadvantages. No single lithotriptor is suitable for all applications and none can meet the goal of fragmenting all calculi while remaining harmless to tissue.


*Diagnostic and Therapeutic Endoscopy*, Vol. 4, pp. 1–7
Reprints available directly from the publisher
Photocopying permitted by license only

© 1997 OPA (Overseas Publishers Association)
Amsterdam B.V. Published in The Netherlands
by Harwood Academic Publishers
Printed in Singapore

### Ureteroscopic Lithotripsy

DEMETRIUS H. BAGLEY

*Department of Urology, Jefferson Medical College, Thomas Jefferson University, Philadelphia, Pennsylvania, USA*

*(Received 12 February 1997; In final form 14 March 1997)*

**There is a wide array of endoscopic lithotriptors presently available. Each of these has its own advantages and disadvantages. No single lithotriptor is suitable for all applications and none can meet the goal of fragmenting all calculi while remaining harmless to tissue.**

*Keywords:* Calculi, Endoscopy, Ureteroscopy, Lasers, Lithotripsy, Urinary tract

### INTRODUCTION

Endoscopic intraureteral lithotripsy has developed as the result of advances in techniques for both ureteroscopy and lithotripsy. Progress has been interdependent and has been successively driven by advances in one field or the other. This progress is apparent as we have moved from the blind ureteroscopic ultrasonic lithotripsy to the present state with small diameter rigid and flexible ureteroscopes and a wide array of lithotriptor devices with very small flexible lithotriptor probes.

Extracorporeal shockwave lithotripsy (ESWL) is often effective for fragmenting urinary calculi. However, endoscopic lithotripsy is superior in managing calculi in certain clinical settings. Impacted and denser stones are harder to fragment with any technique, and often resistant to ESWL. Endoscopic lithotripsy is a method of delivering energy precisely to fragment these denser and impacted calculi successfully. It also allows the simultaneous removal of stone fragments in one setting. The primary goal in developing these techniques remains to provide a mechanism for fragmenting and removing calculi which is fast, effective, and harmless to the normal tissue.

There are several techniques now available for fragmenting calculi through small endoscopes such as ureteroscopes. The benefits, risks, and costs of each of these procedures vary. No technique is ideal for every situation and the advantages of each should be considered when using one or more devices. These lithotriptors include ultrasound, electrohydraulic, lasers and the most recent entries, the impact devices.

### DEVICES AND CLINICAL EFFECTS

### Ultrasonic Lithotripsy

Ultrasonic lithotripsy was first used for fragmenting calculi through ureteroscopes [1]. The larger interchangeable ureteroscopes which had first become available were used for this application. These early ureteroscopes were difficult to use because there was insufficient room in the sheath to have both the telescope and ultrasound probe in place at the same time. The urologist had to remove the telescope while holding the calculus in place with a basket or other retrieval device. The probe was then passed through the channel vacated by the telescope, applied to the calculus, and activated for fragmentation. The pressure of the probe against the stone in the basket could be felt with the urologist's other hand holding the basket. Thus, this was considered a "tactile" technique. Fluoroscopy could not accurately indicate the position of the probe on the calculus. As the lithotriptor removed a portion of the stone, the change in pressure against the stone could be felt. There remained a danger of passing the probe too far and damaging the mucosa.

A major advance was the development of a rigid ureteroscope with an offset eyepiece telescope [2]. This resulted in a straight channel for passing the rigid ultrasound probe. These endoscopes allowed placement of the rigid probe under direct vision to fragment stones. The device was considerably safer, allowing fragmentation under visual control, but it still required a large diameter (approximately 13F) rigid ureteroscope. Both of these techniques use a hollow ultrasound probe through which small fragments of stone could be removed with suction. The irrigant could also be removed through the probe, both cooling the probe and clearing the visual field. Fragmentation with these instruments was rather slow, although very controlled. The probe is effective only at the point where it touches the calculus. It is also difficult to fragment very hard stones with ultrasound.

A solid wire ultrasound probe has also been described [3]. It requires a straight channel significantly larger than the probe itself to leave room for movement with the vibration of the probe. A smaller ureteroscope approximately 9.5F in size can be utilized with this lithotriptor. The most effective fragmentation is obtained by applying the side of the probe to the calculus and is very rapid even for dense stones such as calcium oxalate monohydrate. However, the fragments are not removed simultaneously.

There is limited risk of tissue damage from these devices. Although there was some concern initially over thermal damage, this can be avoided with adequate irrigation. A more common risk is mechanical trauma to the mucosa from the endoscope or the metal probe itself.

### Electrohydraulic Lithotripsy

The electrohydraulic lithotriptor was the earliest nonmechanical endoscopic lithotriptor. It was described for use in the bladder where the larger probes of 7–9F have been used [4]. This device uses a flexible probe with a coaxial tip. A high voltage electrical discharge placed across the tip creates a spark which in turn causes a bubble in liquid medium. The rapid expansion and contraction of this bubble sets up a shockwave which is relatively well tolerated by soft tissue but can fragment a solid calculus.

Electrohydraulic lithotripsy was initially considered dangerous in the ureter because of studies using it blindly in animal ureters demonstrated perforation of the mucosa [5,6]. The development of small diameter EHL probes permitted their use in the ureter. Probes of 3F or less can be passed through the rigid ureteroscopes and these have been downsized now to 1.6–1.9F. These small probes produce a high energy density and provide very effective fragmentation of all but the hardest calculi. Clinical series have demonstrated fragmentation rates of greater than 90% [7–9]. There remains, however, risk of damage from the laterally directed shockwave. This has been minimized with a new design which offers a more directed shockwave [10]. The overall benefit of this design awaits longer clinical series.

### Pulsed Dye Laser

The pulsed dye laser was developed specifically for its ability to fragment calculi [11]. The wavelength (504 nm) produced by the Coumarin dye laser is preferentially absorbed by a yellow crystalline calculus (Table I). Early studies demonstrated that the light of the laser could be delivered along a flexible fiber to be applied directly to a calculus. It gave very effective fragmentation of calcium oxalate dihydrate calculi but was less effective for the dark calcium oxalate monohydrate stones, and was ineffective against cystine stones [12,13]. The device is also very safe since it does not damage the ureter when directly applied to the mucosa. Thus, two of the major benefits of the pulsed dye laser are that it can fragment calculi without damaging the ureter and that the light can be passed through a very small fiber allowing its use through smaller endoscopes.

Maximal energy of the individual laser and the fiber used can determine the effectiveness in fragmentation. The original design of the pulsed dye laser used a 200 micron fiber with a maximum of 60 millijoule pulse. This was often found to be ineffective against the hard calcium oxalate monohydrate stones. When a 320 micron fiber was used with 140 millijoules there was a marked increase in successful fragmentation of even the calcium oxalate monohydrate stones [14,15]. This combination delivered higher energy with more effect against the stone but also with more potentially damaging effect to the ureteral wall [16].

### Alexandrite

The Alexandrite laser is a solid state pulsed laser which can be used with silicon fibers of 250 micron diameter for energy delivery (Table I). The laser has been used *in vitro* and clinically to fragment most types of calculi. Calcium oxalate dihydrate stones are most easily fragmented while the hard calcium oxalate monohydrate and brushite stones are more difficult. Cystine stones are not effectively fragmented. The Alexandrite laser was generally reported to be as effective as the pulsed dye laser for fragmenting urinary calculi [17–20].

In a clinical series, the Alexandrite laser was compared to pneumatic lithotripsy with the Lithoclast. The pneumatic device was more effective for larger and harder calculi and also appeared more user-friendly and cost-effective [21].

Controversy has surrounded the vulnerability of the fiber delivery system during Alexandrite lithotripsy. It has been demonstrated that the fiber itself fragments with very short pulsed lithotripsy (350 ns), embedding pieces of fiber into the ureteral mucosa. A longer pulse duration of 1 μs had no effect on the fiber [22]. Other studies have not observed this problem [23].

TABLE I Laser Lithotriptors

| | Pulsed Dye | Alexandrite | Holmium |
|---|---|---|---|
| Medium | Coumarin dye | Alexandrite crystal | Holmium : Yttrium aluminum-garnet crystal |
| Wavelength | 504 | 750 | 2100 |
| Fibers (μ) | 250–550 | 250–320 | 200–365–550–1000 |
| Pulse-frequency | 1–10 Hz | 1–20 Hz | 5–30 |
| Pulse-duration | 1 or 1.8 μsec | 500 nsec or 1 μsec | 250 μsec |
| Energy/Pulse | 60–200 mJ | 40–100 mJ | 0.5–2.8 J |
| Calculi treated | not cystine Ca ox mono and brushite–difficult | not cystine | all calculi |

### Holmium

The Holmium (Ho:YAG) laser has recently been introduced for lithotripsy. This laser has the capability of fragmenting calculi but can also ablate tissue [24,25]. This solid state laser produces light at a wavelength of 2100 nanometers in a pulsed fashion (Table I). It is very rapidly absorbed in water and thus has a thermal effect. It produces a very small steam bubble which rapidly expands and contracts [26]. It also possibly has a similar effect within the interstices of the stone. It produces fragmentation of any type of calculus with a "pitting" effect. With continued application of the laser, a large defect can be created in a calculus and the shell fragmented. Smaller calculi can be fragmented directly [27–29]. There is also risk of damage to the ureter since the laser can ablate tissue. However, the effect of the laser is confined to the immediate area in front of the fiber and penetration even in tissue is limited to less than 0.5 mm. Therefore the device is safe for use in the ureter when used with appropriate care. Continuous irrigation should be maintained to cool the irrigant and maintain a clear visual field (Fig. 1). The fiber should be applied only to the area to be treated and care should be taken to avoid activating the laser when it is near the mucosa.

Three recent series treating a total of 238 patients have documented the safety and efficacy of lithotripsy with the holmium laser [30–32]. Stones throughout the urinary tract have been treated successfully. The major clinical benefits of the holmium laser for lithotripsy are that all types of calculi, including cystine and brushite, can be fragmented and that there is little movement of the calculus during treatment.

### IMPACT DEVICES

### Lithoclast

The Lithoclast is an impact device which consists of a stainless steel probe placed endoscopically. It is connected to the transducer containing a metal impactor which is driven with compressed air to impact upon the probe, driving it forward to impact in turn upon the calculus. It gives effective and rapid fragmentation of any type of calculus [33]. A rigid impactor tip requires a straight, or very nearly straight, working channel and therefore an offset rigid ureteroscope is required. It can cause significant movement of the calculus. The only risk to the ureter during use appears to be one of mechanical trauma from the endoscope or the impactor device itself.

The Lithoclast has been quite effective in many clinical series throughout the world [34,35]. It appears to be at least as effective as electrohydraulic or pulsed dye or Alexandrite laser lithotripsy [21,36]. The major limitation to the design appears to be the need for a rigid ureteroscope thus limiting the range of application. There is also movement of the stone during fragmentation which often causes retrograde progression of the calculus into the kidney.

The Browne Pneumatic Impactor is an endoscopic impact lithotriptor which utilizes probes of nitinol which can be used even while angled. This device can be used through any ureteroscope including flexible endoscopes [37,38]. The probe is struck by an impact hammer driven by compressed air and in turn strikes the calculus. The BPI has been demonstrated to be safe and very effective for all types of calculi *in vitro* and in animal studies. A preliminary clinical series has demonstrated effectiveness of the device for distal and mid ureteral calculi [39]. Definition of its role will require larger series.

### Choice of Lithotriptor

The selection of an endoscopic lithotriptor can depend on many factors. Of paramount importance is the location of the calculus. For instance stones in the distal ureter can be accessed best with a rigid endoscope. Those within the proximal ureter or intrarenal collecting system can be reached more easily with a flexible ureteroscope [40,41]. Thus, the choice of lithotriptor is limited by the

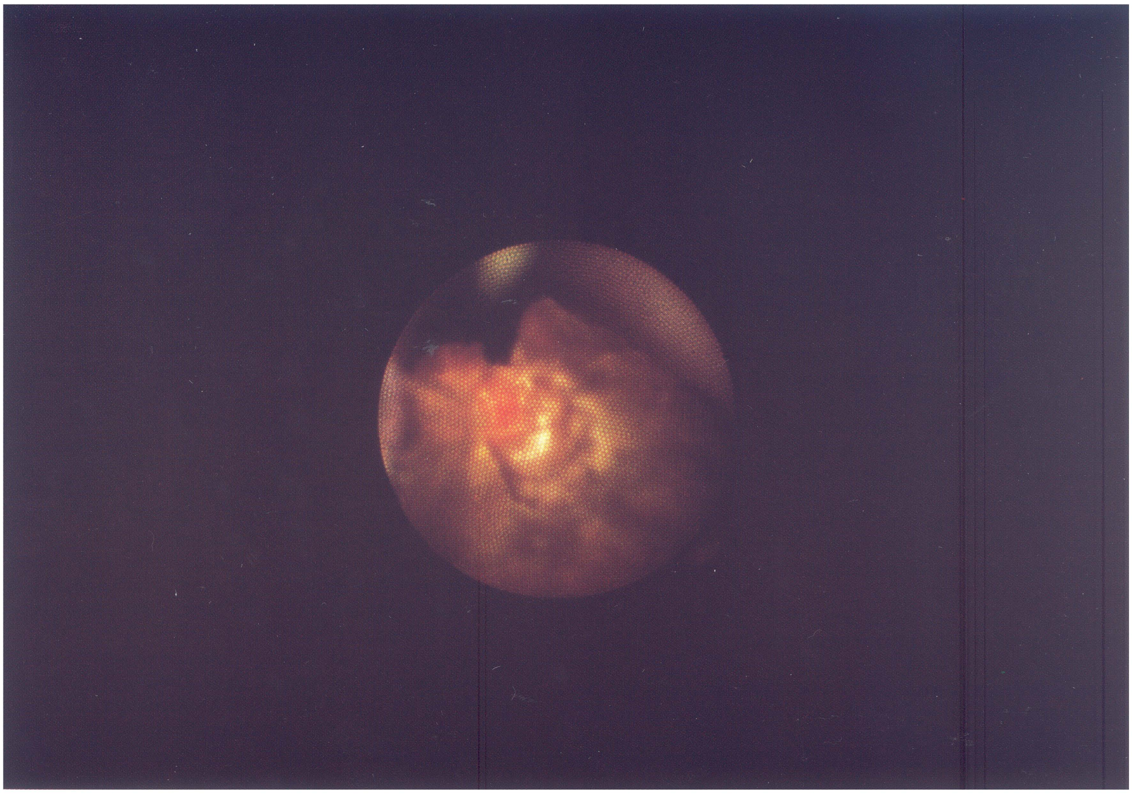

FIGURE 1 The laser fiber is located adjacent to the cavity treated with the Holmium laser lithotriptor.

endoscope which can be employed. For example, it would be impossible to reach a calculus in the mid or lower intrarenal calyces with a rigid ureteroscope needed for an ultrasonic lithotriptor or the Lithoclast. In that case, a flexible ureteroscope using a flexible lithotriptor probe such as the EHL probe or a laser fiber would be the first choice. However, when endoscopy and lithotripsy will be limited to the distal or mid ureter, then a less expensive device than one of the lasers can be used effectively.

The ultrasonic lithotriptor has generally been surpassed in use by one of the other devices since they can be used with much smaller endoscopes and also with the flexible ureteroscopes. The ultrasonic lithotrite remains a first choice for percutaneous nephrostolithotomy because of its ability to remove large volumes of stone. The other lithotriptors are relatively less effective in treating larger calculi and therefore have less of a role in nephroscopic stone removal. They may be used as a first choice through a flexible nephroscope in peripheral portions of the kidney. It is clear that a single endoscopic lithotriptor device is not adequate for all applications.

### Endoscopic Lithotripsy at Other Sites

Lithotripsy is not limited to the ureter but the same lithotriptors and small endoscopes can be employed in other sites. For example the pulsed dye laser has been used for treating biliary stones in the common duct [42]. Electrohydraulic lithotripsy has also been employed but a more limited margin of safety has dampened enthusiasm for its use at that site [43]. We have recently employed the holmium laser for lithotripsy in the gallbladder and common bile duct without complication [44].

### *References*

[1] Huffman, J.L., Bagley, D.H., Schoenberg, H.W. and Lyon, E.S. Transurethral removal of large ureteral and renal pelvic calculi using ureteroscopic ultrasonic lithotripsy. *J. Urol.* 1983; **130**: 31–34.

[2] Huffman, J.L. and Bagley, D.H. The Development of Instrumentation for Transurethral Ureteropyeloscopy. *In. Ureteroscopy.* Huffman J.L., Bagley D.H. and Lyon E.S., eds. Philadelphia, PA, W.B. Saunders, 1988; pp. 1–15.

[3] Boline, G.B. and Belis, J.A. Laser lithotripsy of upper urinary tract calculi after unsuccessful ESWL or ureteroscopy: Comparison with primary lasertripsy. *J. Endourol.* 1993; **7**: 473–476.

[4] Reuter, H.J. Electronic lithotripsy: transurethral treatment of bladder calculi in 50 cases. *J. Urol.* 1970; **104**: 834–838.

[5] Reuter, H.F. and Kern, E. Electronic lithotripsy of ureteral calculi. *J. Urol.* 1973; **110**: 181–183.

[6] Raney, A.M. Electrohydraulic ureterolithotripsy. *Urology* 1978; **12**: 284–288.

[7] Begun, F.P., Jacobs, S.C. and Lawson, R.K. Use of a prototype 3F electrohydraulic electrode with ureteroscopy for treatment of ureteral calculus disease. *J. Urol.* 1988; **139**: 1188–1191.

[8] Denstedt, J.D. and Clayman, R.V. Electrohydraulic lithotripsy of renal and ureteral calculi. *J. Urol.* 1990; **143**: 13–17.

[9] Green, D.F. and Lytton, B. Early experience with direct vision electrohydraulic lithotripsy of ureteral calculi. *J. Urol.* 1985; **133**: 767–770.

[10] Vorreuther, R. New tip design and shock wave pattern of electrohydraulic probes for endoureteral lithotripsy. *J. Endourol.* 1993; **7**: 35–43.

[11] Watson, G.M., Murray, S., Dretler, S.P. and Parrish, J.A. The pulsed dye laser for fragmenting urinary calculi. *J. Urol.* 1987; **138**: 195–198.

[12] Watson, G.M., Landers, B., Nauth-Misir, R. and Wickham, J.E.A. Developments in the ureteroscopes, techniques and accessories associated with laser lithotripsy. *World. J. Urol.* 1993; **11**: 9–25.

[13] Dretler, S.P. An evaluation of ureteral laser lithotripsy: 225 consecutive patients. *J. Urol.* 1990; **143**: 267–272.

[14] Grasso, M. and Bagley, D.H. Endoscopic pulsed dye laser lithotripsy: 159 consecutive cases. *J. Endourol.* 1994; **8**: 25–27.

[15] Vandeursen, H., Pittomvils, G., Boving, R. and Baert, K. High energy pulsed dye laser lithotripsy: Management of ureteral calcium oxalate monohydrate calculi. *J. Urol.* 1991; **145**: 1146–1150.

[16] Wu, T.T., Hsu, T.H., Li, A.F., Chen, M.T. and Chang, L.S. Morphological change in the urothelium after electrohydraulic versus pulsed dye laser lithotripsy. *Br. J. Urol.* 1994; **74**: 685–689.

[17] Weber, H.M., Miller, K., Ruschoff, J., Gschwend, J. and Hautmann, R.E. Alexandrite laser lithotripter in experimental and first clinical application. *J. Endourol.* 1991; **5**: 51–55.

[18] Mattioli, S., Cremona, M., Benaim, G. and Ferrario, A. Lithotripsy with a Q-switched Alexandrite laser system. *Eur. Urol.* 1991; **19**: 233–235.

[19] Benizri, E., Wadey, J., Amiel, J. and Touhol, J. Comparison of 2 pulsed lasers for lithotripsy of ureteral calculi: Report on 154 patients. *J. Urol.* 1993; **150**: 1803–1805.

[20] Jung, P., Wolff, J.M., Mattelaer, P. and Jakse, G. Role of lithotripsy in the management of ureteral calculi: experience with Alexandrite laser system in 232 patients. *J. Endourol.* 1996; **10**: 345:348.

[21] Naqui, S.A.A., Khaliq, M., Zafar, M.N. and Rizvi, S.A.H. Treatment of ureteric stones: Comparison of laser and pneumatic lithotripsy. *Br. J. Urol.* 1994; **74**: 694–698.

[22] Strunge, C.H., Brinkmann, R., Flemming, G. and Engelhardt, R. Interspersion of fragmented fiber splinters into tissue during pulsed Alexandrite laser lithotripsy. *Lasers Surg. Med.* 1991; **11**: 1–5,

[23] Pertus, A.C., Albisu, A., Acha, M., Blasso, M., Llarena, R. and Tanago, J.G. *Invitro* lithotripsy with the Alexandrite laser. *Eur. Urol.* 1992; **22**: 62–63.

[24] Johnson, D.E. Use of the Holmium:YAG (Ho:YAG) laser for treatment of superficial bladder carcinoma. *Lasers Surg. Med.* 1994; **14**: 213–218.

[25] Sayer, J., Johnson, D.E., Price, R.E. and Cromeens, D.M. Ureteral lithotripsy with the Holmium:YAG laser. *J. Clin. Laser Med & Surg.* 1993; **11**: 61–65.

[26] Denstedt, J.D., Razvi, H.A., Sales, J.L. and Eberwein, P. Preliminary experience with Holmium:YAG laser lithotripsy. *J. Endourol.* 1995; **9**: 255–258.

[27] Matsuoka, K., Iida, S., Nakanami, M., Koga, H., Shimada, A., Mihara, T. and Noda, S. Holmium: Yttrium-Aluminum-Garnet laser for endoscopic lithotripsy. *Urology* 1995; **45**: 947–952.

[28] Bagley, D.H. and Erhard, M.J. Use of the Holmium laser in the upper urinary tract. *Tech. Urol.* 1995; **1**: 25–30.

[29] Erhard, M.J. and Bagley, D.H. Urologic applications of the Holmium laser: Preliminary experience. *J. Endourol.* 1995; **9**: 383–386.

[30] Grasso, M. Experience with the Holmium laser as an endoscopic lithotrite: *Urology* 1996; **48**: 199–206.

[31] Razvi, H.A., Denstedt, J.D., Chun, S.S. and Sales, J.L. Intracorporeal lithotripsy with the Holmium:YAG laser. *J. Urol.* 1996; **156**: 912–914.

[32] Shroff, S., Watson, G.M., Parikh, A., Thomas, R., Soonawalla, P.F. and Pope, A. The Holmium:YAG laser for ureteric stones. *Br. J. Urol.* 1996; **78**: 836–839.

[33] Hofbauer, J., Hobarth, K. and Marberger, M. Lithoclast: New and inexpensive mode of intracorporeal lithotripsy. *J. Endourol.* 1992; **6**: 429–432.

[34] Denstedt, J.D., Eberwein, P.M. and Singh, R.R. The Swiss Lithoclast: A new device for intracorporeal lithotripsy. *J. Urol.* 1992; **148**: 1088–1090.

[35] Schulze, H., Haupt, G., Piergiovanni, M., Wisard, M., von Niederhausern, W. and Senge, T. The Swiss Lithoclast: A new device for endoscopic stone disintegration. *J. Urol.* 1993; **149**: 15–18.

[36] Hofbauer, J., Hobarth, K. and Marberger, M. Electrohydraulic versus pneumatic disintegration in the treatment of ureteral stones: a randomized, prospective trial. *J. Urol.* 1995; **153**: 623–625.

[37] Grasso, M., Loisides, P., Beaghler, M. and Bagley, D.H. Treatment of urinary calculi in a porcine/canine model using the Browne Pneumatic Impactor. *Urology* 1994; **44**: 937–941.

[38] Loisides, P., Grasso, M. and Bagley, D.H. Mechanical impactor employing Nitinol probes to fragment human calculi: Fragmentation efficiency with flexible endoscope deflection. *J. Endourol.* 1995; **9**: 371–374.

[39] Tawfiek, E.R., Grasso, M. and Bagley, D.H. Initial use of the Browne Pneumatic Impactor. *J. Endourol.* 1997; **11**(2): 121–124.

[40] Bagley, D.H. Ureteroscopic stone retrieval: Rigid versus flexible endoscopes. *Sem. Urol.* 1994; **12**: 32–38.

[41] Erhard, M., Salwen, J. and Bagley, D.H. Ureteroscopic removal of mid and proximal ureteral calculi. *J. Urol.* 1996; **155**: 38–42.

[42] Grasso, M., Bagley, D.H. and Sullivan, K. Pulsed dye laser lithotripsy: The present state of urologic and biliary applications. *J. Clin. Laser Med. & Surg.* 1991; **9**: 355–359.

[43] Picus, D., Weyman, P.J. and Marx, M.V. Role of percutaneous intracorporeal electrohydraulic lithotripsy in the treatment of biliary tract calculi. *Radiol.* 1989; **170**: 989–993.

[44] Treatment of biliary calculi using Holmium:YAG laser. *J. Endourol.* **10**(1): S133, (Abstract #P9–297), 1996.

